# Development of an AI based automated analysis of pediatric Apple Watch iECGs

**DOI:** 10.3389/fped.2023.1185629

**Published:** 2023-05-23

**Authors:** L. Teich, D. Franke, A. Michaelis, I. Dähnert, R. A. Gebauer, F. Markel, C. Paech

**Affiliations:** ^1^ Department for Pediatric Cardiology, University of Leipzig - Heart Center, Leipzig, Germany; ^2^ Arbeitskreis Integrierte Informationssysteme, Westsächsische Hochschule Zwickau, Zwickau, Saxony, Germany

**Keywords:** Apple Watch, ECG, pediatric cardiology, CNN, smart living

## Abstract

**Introduction:**

The Apple Watch valuably records event-based electrocardiograms (iECG) in children, as shown in recent studies by Paech et al. In contrast to adults, though, the automatic heart rhythm classification of the Apple Watch did not provide satisfactory results in children. Therefore, ECG analysis is limited to interpretation by a pediatric cardiologist. To surmount this difficulty, an artificial intelligence (AI) based algorithm for the automatic interpretation of pediatric Apple Watch iECGs was developed in this study.

**Methods:**

A first AI-based algorithm was designed and trained based on prerecorded and manually classified i.e., labeled iECGs. Afterward the algorithm was evaluated in a prospectively recruited cohort of children at the Leipzig Heart Center. iECG evaluation by the algorithm was compared to the 12-lead-ECG evaluation by a pediatric cardiologist (gold standard). The outcomes were then used to calculate the sensitivity and specificity of the Apple Software and the self-developed AI.

**Results:**

The main features of the newly developed AI algorithm and the rapid development cycle are presented. Forty-eight pediatric patients were enrolled in this study. The AI reached a specificity of 96.7% and a sensitivity of 66.7% for classifying a normal sinus rhythm.

**Conclusion:**

The current study presents a first AI-based algorithm for the automatic heart rhythm classification of pediatric iECGs, and therefore provides the basis for further development of the AI-based iECG analysis in children as soon as more training data are available. More training in the AI algorithm is inevitable to enable the AI-based iECG analysis to work as a medical tool in complex patients.

## 1. Introduction

Recently, smartwatches like the Apple Watch gained popularity and are used by adults and children. These devices offer many features: one of them is the mobile recording of a high-quality iECG. While the recording of an iECG is already possible, an AI-based automated interpretation of the iECG is currently only available for adults and the classification of a minimal amount of rhythms, meaning normal sinus rhythm or atrial fibrillation. In recent studies, Paech et al. and Leroux et al. demonstrated that the Apple Watch valuably records event-based iECGs in children (1–4). Yet, in contrast to adults, the automatic heart rhythm classification of the Apple Watch did not provide satisfactory results in children as the algorithms need to be designed and trained in this group of users. Therefore, this study aimed to design an AI-based algorithm for the automatic high-quality interpretation of pediatric Apple Watch iECGs. In this first step, the created algorithm should identify a sinus rhythm in children. When more data of pediatric arrhythmias become available, the algorithm will be trained, and in the second step, it should detect pediatric arrhythmias.

## 2. Methods

### 2.1. Convolutional neuronal network (CNN) choice

The Scrum team at the West Saxon University of Applied Sciences in Zwickau designed an AI software for us, using a convolutional neuronal network (CNN). The software consists of two parts: an AI-based analysis of the single-beat morphology and the evaluation of the RR interval. The analysis is performed according to age-specific thresholds to ensure it can also be used in children.

Jun et al. presented a CNN model that is well suited for processing ECGs: the model served as a starting point for our development of the model w_ws_c70 (5). This served as our prototype and was compared to the model Xception, which outperforms in accuracy on the ImageNet dataset (6) and is therefore highly effective in image analysis.

Based on the data of about 500 fully labeled pediatric iECGs and the open-source MIT data set, our team trained and optimized these convolutional neuronal networks.

### 2.2. ECG training data

The raw data consisted of 500 labeled 30 s long pediatric iECGs. These data were acquired at the Department for Pediatric Cardiology at the Leipzig Heart Center as part of a previous study (2). They were registered using an Apple Watch and an iPhone. After recording, the data were available on the paired iPhone and could be sent to another device via the iPhone's Health app profile menu under “Export all health data”. After being exported from the iPhone, the data were available as a.csv file (7–9).

For the analysis via CNNs, the data had to be converted into a two-dimensional form. Matplotlib and OpenCV programs were used to split the iECG signals into images of individual beats following a special algorithm (10). The programs designed images in different sizes for both CNNs: for the CNN w_ws_c70 images with 128 × 128 pixels and for the Xception images with 299 × 299 pixels.

**Figure 1:**
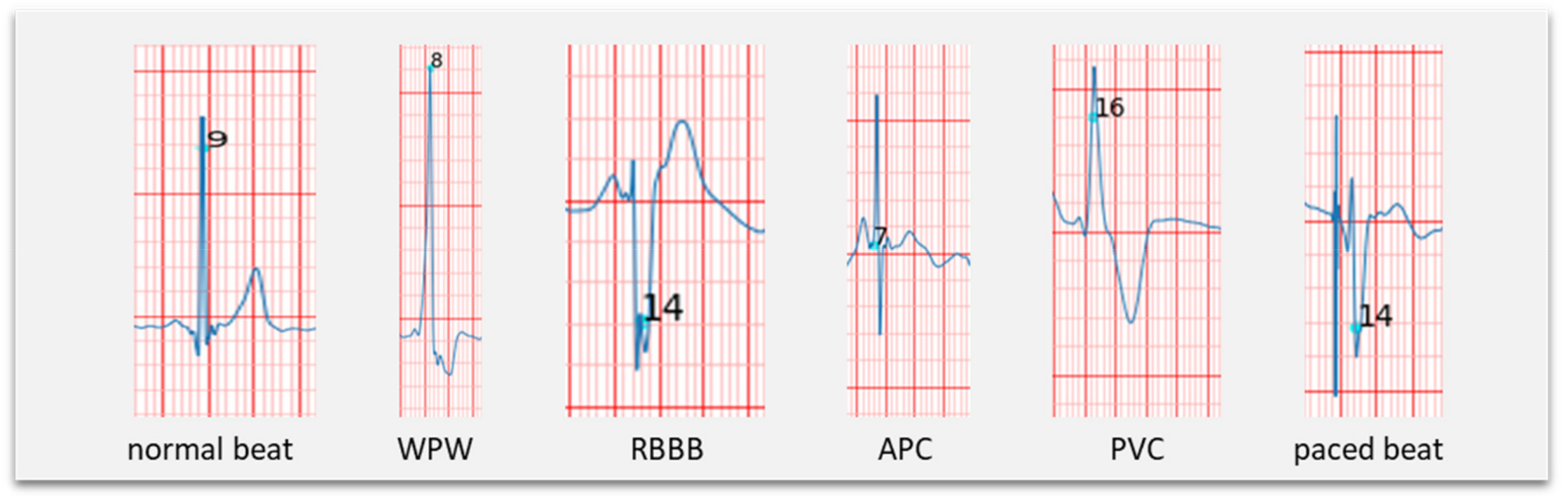
Examples of different beat morphologies.

After this procedure, the following data became available: 19,320 regular beats (N), 2941 right bundle branch block beats (RBBB, R), 813 pacemaker beats (P), 238 Wolff-Parkinson-White (WPW, W) beats, ten premature ventricular contractions (PVC, V), and five premature atrial contractions (APC, S). Figure 1 shows examples of those beat morphologies.

### 2.3. Data setup and augmentation

The individual beats were then separately assigned to their classes. Due to the small number of examples in the classes WPW and APC, they were excluded.

Since a CNN needs large amounts of training data for good performance, two methods for data augmentation can be used to increase the amount of training data. While data for the premature ventricular contractions class could be extended with examples from the open-source MIT-BIH dataset (11), including 7,125 PVC beats, data for the pacemaker beats could be extended using the augmentation method: Nine sections with the size of two-thirds of the original image are cut out, enlarged to the target size and result in ten similar images.

Subsequently, the same number of images was selected for all classes to obtain balanced training data.

In the last step, this raw data was divided into training, validation, and test data for training the models.

### 2.4. Training of the two different models

The CNNs differ in their architecture: While the w_ws_c70 network uses a linear structure in which the individual layers are traversed one after the other, the architecture of Xception is more complex, with three flows being traversed one after the other and repeated if necessary (12). The result of the training runs with the Xception model was the model Xveption_v8. This was the run with the best performance.

**Figure 2:**
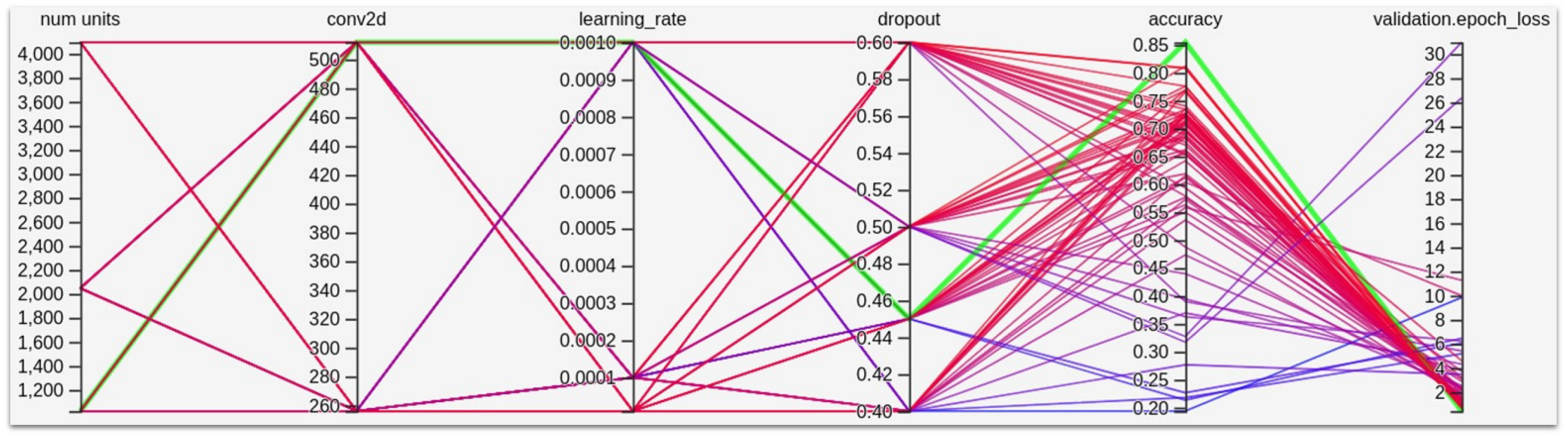
Hyperparameter optimization.

Training of the models was achieved with different methods: optimization of hyperparameters, number of neurons in the last dense layer, size of the last conv2d layer, learning rate, or dropout. The Tensorboard program helped to perform an analysis comparing potentially relevant parameter combinations. Optimization of hyperparameters is demonstrated in Figure 2: The best setting is highlighted in green.

Dropout is a method for regulating models and works by removing a defined number of connections between the layers, leading to redundant representations being learned.

In order to achieve a better generalization of the models, two percent of the pixels were randomly colored black, which is called “Salt and Pepper Noise”. If 70% of their training images had this “noise”, CNN models produced the best results.

### 2.5. iECG examination workflow

**Figure 3:**
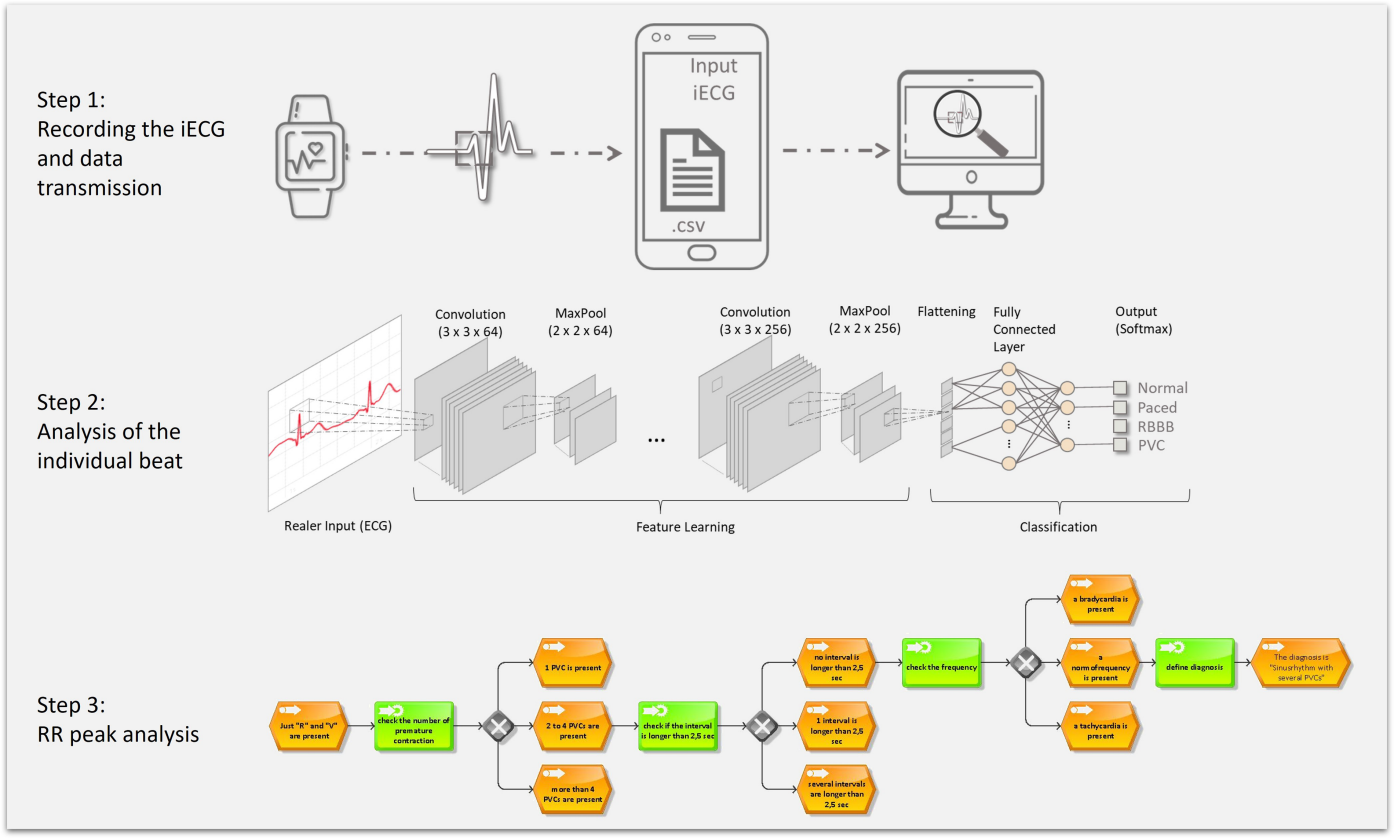
Baseline examination workflow used to evaluate the final AI-based algorithm.

After training, the algorithm had to be tested. The workflow of the measurement up to the final diagnosis is shown in Figure 3.

First, the watch is attached to the wrist, and the patient is asked to record an iECG, automatically transferred to the paired iPhone. The iECG is then sent to the evaluation computer. The first five seconds are removed in iECGs with strong artifacts at the beginning. There was a higher chance of artifacts in younger patients especially in neonates. This difficulty was surmounted by the cutting of the ECG. In the first few seconds motion artifacts were much more prevalent but the potential removal of the first five seconds lead to good results. We decided to remove these few seconds based on the quality of the ECG because they were not related to the heart rhythm but mainly to the patients age.

Step 2 illustrates the analysis: A *segmenter* captures all R-peaks and cuts the iECG into pieces, each containing a single cardiac cycle. Each of these frames is subsequently evaluated by the trained AI and classified into a beat morphology category. The result is then assigned to a specific decision tree, e.g., “normal beat with PVC”. Step 3 demonstrates the RR interval analysis based on a decision tree following an “if, then” structure. Influences include the existing beat morphologies, the RR intervals, and the patient's age. After going through these different criteria within the decision tree, a diagnosis is available.

After the testing data were obtained, the chief investigator made the final evaluation. The results of those tests are shown in Table 1 along with the results from Apple.

**Table 1:** Results of the final evaluation.

Apple Watch	Predicted positive	Predicted negative
Pathology	7	11
Normal sinus rhythm	7	23
Sensitivity: 38.9%	Specificity: 76.7%	
w_ws_c70	Predicted positive	Predicted negative
Pathology	9	9
Normal sinus rhythm	0	30
Sensitivity: 50.0%	Specificity: 100%	
Xception_v8	Predicted positive	Predicted negative
Pathology	12	6
Normal sinus rhythm	1	29
Sensitivity: 66.7%	Specificity: 96.7%	

### 2.6. Patient collective

This prospective, single-arm study included pediatric patients of the outpatient clinic of the Leipzig Heart Center, Department for pediatric cardiology. Children with no (e.g., Syncope), simple (e.g., ventricular septal defect), and complex congenital heart disease (e.g., Ebstein's anomaly) were incorporated. After the informed consent of the parents was given, a single-lead- iECG was obtained using the Apple Watch Series 6. Simultaneously a 12-lead-ECG was recorded as a gold standard to compare the accuracy of the analysis afterward. The exclusion criterion was the refusal to give informed consent.

The reproducibility is given but the research seams to be limited to special facilities. Otherwise, the needed number of pediatric patients with cardiac arrhythmias will not be able to be found due to the low prevalence of those arrythmias.

### 2.7. Recordings

The Apple Watch was placed on the left wrist, and a finger of the right hand pressed the “digital crown”. If this wasn’t possible, the recording positions were changed. Due to motion artifacts, one recording had to be excluded.

The iECGs have a length of 30 s each and were recorded at a rate of 512 hertz, with the individual readings measured in micro-volts (µV).

The 12-lead ECGs were recorded using a Nihon Kohden Cardiofax M ECG-2,350. The writing speed was set to 50 mm/s.

### 2.8. Measurements

The single-lead-iECG was recorded by nurses and an assistant that were instructed beforehand. Nurses measured the 12-lead-ECG, and the analysis and heart rhythm classification was done by two pediatric cardiologists blinded to the patient's data apart from the patient's age. The recordings were performed consecutive within a few minutes. Due to motion artefacts the recordings could not be performed at the same time. Afterwards all ECGs were reviewed by an experienced pediatric cardiologist and no significant change in heart rhythm could be detected.

### 2.9. Statistics

Statistical analysis was performed using Microsoft Excel (Version 2,108) and SPSS (Version 28.0.1.0). The sensitivity and specificity of the different self-developed AI analyzation models and the Apple Watch were compared. In addition, the significance level was calculated using McNemar’s test on paired nominal data.

## 3. Results

Overall, 48 patients were enrolled in this study. The data was collected in two months with the goal of testing the newly developed software. Table 2 shows the patients' characteristics.

**Table 2:** Patients’ characteristics.

Patients’ characteristics (*n* = 48)	Median	Range
Age (years)	9.88	0–17.92
Gender (1 = female; 2 = male)	male = 25/female =	23
Weight (kg)	32.25	3.72–91.70
Height (cm)	131.75	52–181
Congenital heart disease (0 = none; 1 = simple; 2 = complex)	0 = 6/1 = 22/2 =	20

**Figure 4:**
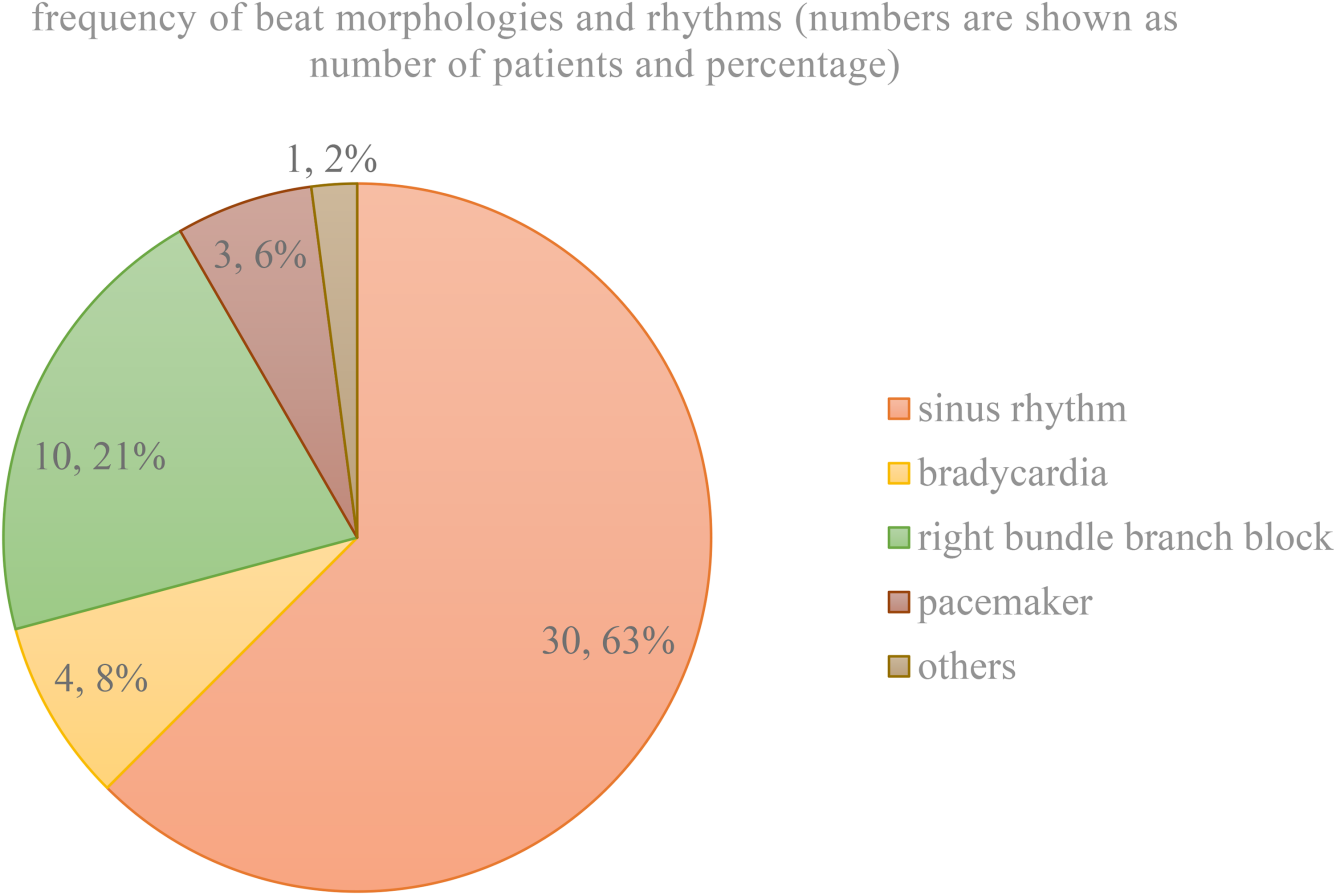
Frequency of beat morphologies and rhythm recorded.

Figure 4 shows the frequency of beat morphologies and rhythm prevalent in the 48 iECGs.

The heart rhythm classification of the 12-lead-ECG by a pediatric cardiologist was set as the gold standard. ECGs were regarded as *healthy,* if a sinus rhythm with narrow QRS complexes was prevalent. All other ECGs were classified as *pathological*, including the appearance of bradycardia or right bundle branch blocks. All tests that correctly identified the pathology were counted as *predicted positive*. All tests that diagnosed a sinus rhythm or a false pathology were counted as *predicted negative*. Both, the self-developed software and the original software of Apple occasionally showed the diagnosis inconclusive. This diagnosis was treated as false by the chief investigator. Table 1 shows the results of the final evaluation.

The different CNNs, w_ws_c70, Xception_v8 and others, were used to identify the prevalent beat morphology. The results varied in some iECGs due to the different structure and training which lead to a different conclusion although the same decision tree was used afterwards. Those results of the different CNNs are shown in the study and illustrate the capability of the various CNNs.

We could demonstrate a significant difference (*p* = 0.004) between the results of the original Apple-designed algorithm that reached a sensitivity of 38.9% and a specificity of 76.7%, and the best algorithm developed by this team that achieved a sensitivity of 66.7% and a specificity of 96.7%. The research if there was any difference in sensitivity or specificity based on age of the patient was not conducted. The number of patients in some age categories were so small that a calculation of sensitivity and specificity could not deliver significant results.

## 4. Discussion

This study demonstrates a first and successful development of an AI-based algorithm for the high-quality automatic classification of pediatric Apple Watch iECGs with regard to the presence of a normal sinus rhythm in differentiation from pathologic heart rhythms. The currently presented data show the potential of developing a fully versatile algorithm specifically designed for pediatric iECGs with the availability of bigger data sets.

Along with the progressive emergence of smart devices like the Apple Watch, people can monitor their health semi-professionally (13–16). While smartwatches have collected data for at least a decade, an interpretation is only possible with the help of newer AI-based algorithms. However, these AI-based automatically interpreted data are mainly restricted to adults. Two main reasons are: the lower availability of pediatric data and the possibility of opening a Google or Apple account only with a certain minimum age.

In this study, the authors opted for a relatively new workflow in the sense of a scrum team. This enabled the study team to present solutions after only four months of development (17). The advantages of designing a program with Scrum are: economic time management, high effectiveness, and flexibility (the product owner can test the newly developed software after each development cycle, and changes can be requested throughout the designing process). Due to the requirements of a huge number of complete data sets, only a part of the heart rhythm classification tool could be initialized using a novel AI algorithm, while a second part of the application still works as a rule-based model.

The newly developed algorithm with the model Xception_v8 showed a specificity of 96%, while the sensitivity of this program was relatively low at 66.7%. The AI does not recognize certain pathologies, yet, mainly due to two facts: The amount of datasets with pathologies was relatively low, and some pathologies might not be detected due to the mode of a single-lead-iECG. The first problem can be fixed with further training and more data from a more extensive database (18). The second difficulty could be matched with more leads, maybe even new ones. But the AI securely diagnoses a normal sinus rhythm in differentiation from a pathologic heart rhythm and may represent valuable information to, for example, parents of a child with suspected arrhythmia.

Finally, further research with the goal of improving the algorithm and enforce the development of a completely AI-based heart rhythm classification in children is currently planned and under development. Further studies and enhanced data collection will be needed to present a fully functioning and reliable algorithm, for example, in the form of an app.

## 5. Conclusion

The current study presents a first AI-based algorithm for the automatic heart rhythm classification of pediatric iECGs. It furthermore represents a basis for further development of the AI-based iECG analysis in children as soon as more training data are available. Further improvement may help to transfer this tool from a lifestyle health product into a reliable medical tool.

## Data Availability

The datasets presented in this article are not readily available because in the dataset, there are mainly data that were collected on children. Publication of the data as a dataset outside of the publication was not approved. Requests to access the datasets should be directed to lt17vado@studserv.uni-leipzig.de.

## 6. Limitations

This study is limited by the limited training data on pediatric iECGs and therewith limiting the study to a proof of concept. The results must be validated by further research in a bigger population, with a bigger number of pathologies and a healthy control group.
